# Supplementary material for: Accuracy Assessment of the Planar Morphology of Valley Bank Gullies Extracted with High Resolution Remote Sensing Imagery on the Loess Plateau, China
Source: Int J Environ Res Public Health. 2019 Jan 28;16(3):369. doi: 10.3390/ijerph16030369 (PMC6388579; doi:10.3390/ijerph16030369)
Supplement: Supplementary file 1 [file ijerph-16-00369-s001.pdf]

Supplementary Table. Available gully area of the two methods under unitary time, labor and cost.

Items	Notes	Field survey using differential global positioning system (dGPS)	Visual interpretation from image
Field time, day	For positioning, calibration and measuring.	20	-
Process time, day	For processing the field data, interpreting image and processing the interpreted gully edges.	10	34 (24 days for interpreting and 10 days for processing)
Unitary total time, min/m^2	Total time = field time + process time.	1.8	2.0
Labor in the field, person	Including five operators (one for adjusting and looking after the base station, the other four divided into two teams for measuring in the forenoon and afternoon, respectively) and one driver.	6	1
Labor in processing, person	-	1	1
Unitary total labor requirements, person/m^2	Total labor = field labor + processing labor.	0.4	0.1
Material costs, $	For purchasing the dGPS or satellite image. The actual area of study watershed is 10. 54 km^2, but the image could only be bought with the minimum enclosing rectangle of the watershed (31 km^2). The price for image was 21.83 $/km^2.	17,467[a]	676.86
Field costs, $	Including the items below.	3,463.82	-
Food and drink, $	Budget (2.91 $/day) by number of person by working time (day). The similar below.	2.91*6*20 = 349.34	-
Accommodation, $	1.75 $/night for living in the experimental station of our institute, 7.28 $/night at least if living in hotels.	1.75*6*20 =210 (7.28*6*20 = 873.36 If Living in Hotel)	-
Travelling allowance, $	This is for the operators, and that for the driver is contained in the transportation costs.	11.64*5*20 = 1,164.48	-
Transportation, $	One car with seven seats costing 87 $/day at least.	87*20 = 1,740	-

Items	Notes	Field survey using differential global positioning system (dGPS)	Visual interpretation from image
Unitary cost, $/m^2$	-	0.14	0.03
Available gully area, m^2/(min·person·dollar)		10 (8 if living in hotel)	167

Note: [a] The cost of buying dGPS ($17,467) was not contained in the calculation of unitary cost, due to the high reusability and expensive price of the dGPS. All the costs were estimated with the market prices at that time and were transformed into U.S. dollar ($) based on the exchange rate (1 dollar ≈ 6. 87 RMB) on December 9, 2018.
